# Changes in the profile of adults diagnosed as autistic since 2010: population-based studies in the UK and Sweden

**DOI:** 10.1136/bmjment-2026-302983

**Published:** 2026-11-03

**Authors:** Aws Sadik, Michael Lundberg, Golam M Khandaker, Antonio F Pardiñas, Brian K Lee, Paul Madley-Dowd, Cecilia Magnusson, Dheeraj Rai

**Affiliations:** 1 MRC Integrative Epidemiology Unit, University of Bristol, Bristol, UK; 2 Avon and Wiltshire Mental Health Partnership NHS Trust, Bath, UK; 3 Centre for Academic Mental Health, University of Bristol, Bristol, UK; 4 NIHR Bristol Biomedical Research Centre, Bristol NHS Foundation Trust, Bristol, UK; 5 Department of Global Public Health, Karolinska Institutet, Stockholm, Sweden; 6 NIHR Bristol Clinical Research Facility, Bristol NHS Foundation Trust, Bristol, UK; 7 Centre for Neuropsychiatric Genetics and Genomics, Cardiff University, Cardiff, UK; 8 A.J. Drexel Autism Institute, Drexel University, Philadelphia, Pennsylvania, USA; 9 Department of Epidemiology and Biostatistics, Drexel University Dornsife School of Public Health, Philadelphia, Pennsylvania, USA; 10 Centre for Epidemiology and Community Medicine, Region Stockholm, Stockholm, Sweden; 11 NIHR Applied Health Collaboration South West, University of Bristol, Bristol, UK

**Keywords:** Neurodevelopmental Disorders, Mental Health, Socioeconomic Factors

## Abstract

**Background:**

Autism diagnoses have risen consistently over time. Characterising the backgrounds of adults being diagnosed with autism can help to clarify why.

**Objective:**

To understand if sociodemographic and neuropsychiatric characteristics of adults diagnosed with autism in the UK and Sweden have changed since 2010.

**Methods:**

Using UK primary care records from 2010 to 2023 and Swedish population-wide register linkages from 2010 to 2021, we calculated annual age-standardised incidence and prevalence of adult autism diagnoses within different sociodemographic groups. We also calculated annual age-standardised proportion of adults with prior records of other neuropsychiatric conditions or medications by autism diagnosis status.

**Findings:**

In the UK, 24 537 039 adults contributed to the study, including 141 119 with an autism diagnosis. In Sweden, 9 096 874 adults contributed, including 100 817 with an autism diagnosis. Incident (first recorded) adult autism diagnoses were consistently higher in Sweden than the UK; however, incidence increased rapidly in the UK after 2020. Incident diagnoses increased fastest for 16–20 year-olds and females in both nations, as well as people in white ethnic groups in the UK and people with Swedish-born parents in Sweden. Over time, there has been a consistent decline in the proportion of autistic adults with a prior diagnosis of epilepsy, psychosis and intellectual disability and an increase in the proportion with a prior diagnosis of attention-deficit hyperactivity disorder, anxiety, depression and several other mental illnesses. For example, in the UK between 2010 and 2023, a history of epilepsy among adults with newly recorded autism decreased from 10% (95% CI 7.6% to 13%) to 4% (95% CI 3.6% to 4.5%), while a history of anxiety increased from 28.7% (95% CI 24.4% to 33.6%) to 58.3% (95% CI 56.6% to 60.1%). Trends were broadly consistent between females and males.

**Conclusions:**

The characteristics of individuals diagnosed as autistic have changed dramatically since 2010 in both the UK and Sweden.

**Clinical implications:**

Service design and clinical practice around autistic healthcare should account for inequitable distribution of autism diagnoses in society, the diagnostic complexity associated with other mental health conditions and apparent changes in the average health needs of autistic groups. The extent to which these changes indicate nuanced recognition of autism or broadening of diagnostic practice needs investigation.

**WHAT IS ALREADY KNOWN ON THIS TOPIC**

- Autism diagnoses have increased internationally, with prevalence in high-income countries starting to overtake estimates from population survey-based methods. It is unclear how much this reflects improved understanding of nuanced presentations or broadening of the medical concept of autism beyond coherent boundaries.

**WHAT THIS STUDY ADDS**

- The social and medical backgrounds of people receiving autism diagnoses have changed dramatically over time, particularly for females in the UK.

**HOW THIS STUDY MIGHT AFFECT RESEARCH, PRACTICE OR POLICY**

- In designing diagnostic and postdiagnostic services for autistic adults, policymakers should account for the findings that: (1) diagnoses are inequitably distributed in society, (2) other mental health conditions are adding complexity to the diagnostic process and (3) the average health needs of autistic groups are evolving.

## Introduction

 Autism is a neurodevelopmental condition characterised by social communication difficulties and restricted, repetitive patterns of behaviours and interests. The number of people being diagnosed as autistic has increased worldwide in recent decades,^1–3^ yet the distribution of autistic features across the population appears to have remained stable.^4–7^ In the UK and Sweden, there appeared to have been a greater increase in diagnoses of autism without language or cognitive delays compared with autism with language or cognitive delays.^1 2^ There is also some evidence in Sweden that more recently diagnosed autistic children have lower autism symptom scores on average than children diagnosed in the past.^8^ These findings suggest that there has been a general change in the profile of people being diagnosed with autism. This may be an appropriate shift caused by increased public awareness of autism, reduced stigma,^9^ greater resources available for diagnosis and professional appreciation of more nuanced features, such as autistic presentations among women.^10^ However, it has also been suggested that diagnostic practice may have become overinclusive^11^ possibly through changing views about the level of impairment associated with autistic traits,^12^ misattribution of symptoms of mental health conditions, such as social anxiety to autism, or an autism diagnosis being seen as a way to unlock support for vulnerable individuals who would otherwise not be served by overstretched health, education or social care services.^13^ From a healthcare perspective, overinclusive diagnostic practice could promote a pathologisation of self-identity, shift attention away from modifiable causes of distress and potentially lead to misallocation of resources.

To understand why autism diagnoses are increasing, we can look to the sociodemographic and neuropsychiatric backgrounds of those being diagnosed. Studies in the USA have generally reported the prevalence of autism to be in higher families with higher socioeconomic and educational status.^14–16^ However, the inverse has been found in regions with more equitable healthcare systems, including Europe,^17–19^ Canada^20^ and Japan.^21^ If increasing diagnoses are focused within certain sociodemographic groups, then cultural trends or healthcare disparities may be potential explanations. Neuropsychiatric diagnoses and medication histories offer added insight by illustrating diagnostic complexity and potential support needs.^22^ For example, intellectual disability (ID) can be a marker of functional impairment, and ‘boundary conditions’ such as social anxiety disorder can be difficult to differentiate from autism. Sociodemographic patterns and healthcare practice can vary between nations. Therefore, comparing the consistency of trends across nations with different social contexts and healthcare systems can help indicate whether trends are generalisable or localised phenomena.

Using UK primary care records up to 2023 and Swedish population-wide register linkages up to 2021, we studied the following questions: (1) Has there been a change in the sociodemographic patterning of incident and prevalent autism diagnoses in adults? (2) Has there been a change in the frequency that other neuropsychiatric conditions are recorded in those with incident and prevalent autism diagnoses? (3) Have these trends varied by sex? (4) Are trends consistent in direction and magnitude across the UK and Sweden? Both incident and prevalent diagnoses were assessed because incident diagnoses offered contemporaneous reflections of diagnostic trends, while prevalent diagnoses integrate information from childhood and prior calendar years to outline current public health needs.

## Methods

### Data sources

In the UK, we used the March 2024 extract of Clinical Practice Research Datalink (CPRD) Aurum. CPRD Aurum is a database containing routinely collected electronic health records from primary care practices that use EMIS Web.^23^ The March 2024 extract included over 47 413 279 research acceptable patients (as defined by CPRD) from 1784 general practices in the UK, including over 16 million patients currently contributing data with a median follow-up time of 9.55 years among current contributors.^24^ 35 240 366 of these patients were eligible for linkages, such as patient postcode-level Index of Multiple Deprivation (IMD) quintiles, at the time of the release.^24^ CPRD Aurum has been shown to be broadly representative of the English population in geographical spread and socioeconomic status (SES).^23^

In Sweden, we used linked longitudinal, total population registers, including 13 345 950 people: the Total Population Register (TPR),^25^ the National Patient Register (NPR),^26^ the Cause of Death Register^27^ and the Longitudinal Integration Database for Occupational Research (LISA).^28^ The NPR has coverage of psychiatric admissions and emergency department visits in Sweden from 1973, all somatic inpatient contacts from 1987 and outpatient secondary care since 2001.^26^ The whole population in Sweden is eligible to access specialist care, and it is mandatory for all secondary care physicians to deliver data to the NPR.^26^

### Patient and public involvement

Two autistic advisors helped through meetings starting before the conception of the study to shape the research questions and interpret findings. This included highlighting conditions and medications of particular interest. A third autistic advisor reviewed and provided comments on the manuscript. They continue to contribute to developing dissemination plans and priorities for future research.

### Study population

People entered the study on the date that all of the following criteria were met: 1 January 2010, their 16th birthday and, in the UK, at least 1 year of registration at a general practice. Follow-up ended at the earliest of the following: death, no longer registered in a practice (UK), migration out (Sweden) or study end date (31 December 2023 in the UK and 31 December 2021 in Sweden). In the analysis of incident (first recorded) diagnosis rates, participants did not contribute follow-up time after a diagnosis of autism. For the analysis of trends in neuropsychiatric history prior to a new autism diagnosis, matching was performed to ensure alignment of reference dates between index adults and comparators. Up to four age-, sex- and practice (UK)- or municipality (Sweden)-matched adults without a recorded autism diagnosis at the index date were selected for each adult with a first recorded autism diagnosis. For the analysis of trends in neuropsychiatric history among all autistic adults (ie, prevalent autism diagnoses), on 31 December for each calendar year, we stratified the whole study population by whether they had ever received an autism diagnosis by that date.

### Sociodemographic and neuropsychiatric variables

In the English dataset, year of birth, sex and geographical region were defined by CPRD. An ethnic group variable with six potential values (mixed, white, South Asian, black, other or unknown) was prepared by identifying published code lists^29 30^ and searching for additional SNOMED, Read and EMIS codes in the CPRD Code Browser. Patient postcode-linked 2019 English IMD quintiles were used as a measure of area-level deprivation.^31^ In the Swedish dataset, sex, date of birth, birth country and parents’ birth countries were obtained from the TPR; parental household income quintiles and education levels were obtained from the LISA. Birth country was coded as Sweden, not Sweden or unknown. Parental countries of birth were coded as both Sweden, neither Sweden, Sweden/not Sweden or unknown. Parental household income quintiles were taken on the index person’s birth year, so that the index person’s autism status could not impact the income variable. Parental education levels were coded as compulsory, upper secondary, further education or unknown and were taken on the year the index person turned 15, because this variable was only available from 1990. In primary analyses, the highest level from either parent was used for household income and education levels. Individual parent values (ie, mother or father) were used in sensitivity analyses. The term *sex* is used throughout this study because the variable labelled *gender* in CPRD data generally reflects the sex recorded at birth, based on biological characteristics, and sex at birth is recorded in the Swedish TPR.

We identified each person’s first record of the following neuropsychiatric conditions: autism, ID, attention-deficit hyperactivity disorder (ADHD), epilepsy, migraines/headaches, anxiety, depression, psychotic disorders, manic/bipolar disorders, obsessive-compulsive disorder and related conditions, stress-related conditions (including post-traumatic stress disorder), eating disorders, personality disorders, suicidality/self-harm, sleep disorders and chronic fatigue. We also identified each person’s first recorded prescription (UK) or dispensation (Sweden) of seven medication classes: antipsychotics, antidepressants, antiseizure medications, opioids, gabapentinoids, melatonin and benzodiazepines/z-drugs (sedatives). In CPRD, SNOMED, Read and EMIS codes for psychiatric conditions and medications were identified through code lists developed by AS, a clinician, and checked against previous code lists,^32^ with any disagreements resolved in discussion with other clinicians in the team. Previously published code lists were used for epilepsy, migraine/headache and chronic fatigue.^32^ In Swedish registers, health conditions and medications were identified by International Classification of Diseases (ICD) version 8, 9 and 10 codes and Anatomical Therapeutic Chemical classification codes, respectively, using lists developed by AS and ML. We studied both conditions and medications because these are each imperfect but complementary approaches to understanding neuropsychiatric history. Mental health categories were chosen to broadly reflect ICD-11 and Diagnostic and Statistical Manual 5 chapters, which can be mapped onto codes available in CPRD and the NPR. Suicidality/self-harm, migraine/headache and chronic fatigue were also chosen as neuropsychiatric characteristics due to their reported importance to the autistic community and acceptable levels of recording in the datasets. Diagnoses required one record to allow dates to be interpreted as the first record of a condition.

### Statistical analyses

In each nation, the annual incidence of autism diagnoses was calculated by dividing the number of adults with a first record of autism by the person-years of follow-up among those eligible to have a first autism diagnosis. The annual prevalence of autism diagnoses was calculated on 31 December of each year by dividing the number of adults with a prior autism diagnosis by the total number of adults in the study on that date. These calculations were repeated with the population stratified by age group (16–20, 21–25, 26–30, 31–40, 41–65 years old). Annual incident and prevalent autism diagnosis rates for each sex and sociodemographic group were age standardised to aid comparability between strata and calendar years. We performed direct standardisation using the mid-2022 age distribution of adults aged 16–65 years in the UK^33^ (online supplemental table S1), due to the low numbers of diagnoses in over 65s. Parental information in Sweden was unavailable for those born prior to 1973, so these analyses were restricted to 16–35 years old to enable use of a consistent standard population from 2010 to the study end. People with unknown values for sociodemographic variables were included in the analyses. For clarity of presentation, the results for the ‘unknown’ strata for each variable are presented in the online supplemental tables rather than primary figures. We also statistically evaluated sociodemographic differences in trends of the rates of new records of autism diagnosis on the multiplicative scale through interaction terms in Poisson regression models (detailed in the online supplemental methods).

For incident analyses, after matching adults with a first record of autism with up to four comparators each, we reported counts and percentages for their distribution of index dates by time period (the autistic person’s diagnosis date: 2010–2014, 2015–2019 or 2020 to study end) and their distribution by sex, age group at diagnosis and aforementioned sociodemographic variables. We also reported the median and IQR for the length of time registered in the dataset prior to the date of study inclusion.

For each condition and medication of interest, in each calendar year we calculated the age-standardised proportion of adults with a first recorded autism diagnosis and comparators who had ever had that condition/medication recorded by the index autism diagnosis date. It is possible that these conditions/medications were recorded before or during the person’s autism diagnostic journey. We performed direct standardisation using the empirical age distribution of adults with a first recorded autism diagnosis from 2010 to 2021 in both datasets combined (online supplemental table S2). We used 5-year age bands from 16 years old to 65 years old for standardisation as diagnoses became sparse beyond 65 years old. We repeated these analyses for all autistic adults by using 31 December as the index date for each calendar year and stratifying all adults in the whole study population by whether they had ever received an autism diagnosis before that date. We used the empirical age distribution of autistic adults across the datasets in 2021, restricted to 16–65 year-olds, to ensure that there were autistic adults at each age for each calendar year (online supplemental table S3). For each condition/medication and comparator group, we also calculated both the absolute and relative changes in age-standardised proportion from 2010 to 2021. Each analysis was repeated with the groups stratified by sex.

CIs for age-standardised incidence, prevalence and proportions were calculated using Byar’s method^34^ with Dobson method adjustment,^35^ consistent with methods recommended by the UK Department of Health and Social Care.^36^ CIs for absolute differences and rate ratios were calculated using the Agresti/Caffo method^37^ and the Wald/Katz-log method,^38^ respectively.

The population, denominator, index date, standard population and statistical measure for each analysis are summarised in online supplemental table S4.

## Results

### Adults with a first recorded autism diagnosis (incident autism diagnoses)

The UK data included 155 872 147 person-years of follow-up time between 2010 and 2023, with 38 858 receiving a new autism diagnosis during this time. The Swedish dataset included 87 155 893 person-years of follow-up time between 2010 and 2021, with 50 239 receiving a new autism diagnosis during this time. Figure 1 presents the changes over time in first recorded autism diagnoses by sociodemographic group, with underlying values present in online supplemental tables S5–S7. Rates of first recorded autism diagnosis were consistently lower in the UK than Sweden; however, rates appeared to grow faster in the UK than Sweden over time, particularly after 2019. In the UK, rates increased from 0.6 diagnoses per 10 000 person-years in 2010 to 6.4 diagnoses per 10 000 person-years in 2023. In Sweden, they increased from 4.3 diagnoses per 10 000 person-years in 2010 to 6.5 diagnoses per 10 000 person-years in 2021. In both nations, rates of first recorded autism diagnosis were highest and increased fastest for 16–20 year-olds than other age groups, increasing from 3.1 diagnoses per 10 000 person-years in 2010 to 36.4 in 2023 in the UK, and from 16.5 diagnoses per 10 000 person-years in 2010 to 36.1 in 2021 in Sweden. Rates of first recorded autism diagnosis among males were higher than females in 2010, but were higher among females than males by the end of the study period: 10.2 and 6.7 diagnoses per 10 000 person-years among females and males in the UK in 2023, respectively, and 9.4 and 8 diagnoses per 10 000 person-years among females and males in Sweden in 2021, respectively.

**Figure 1:**
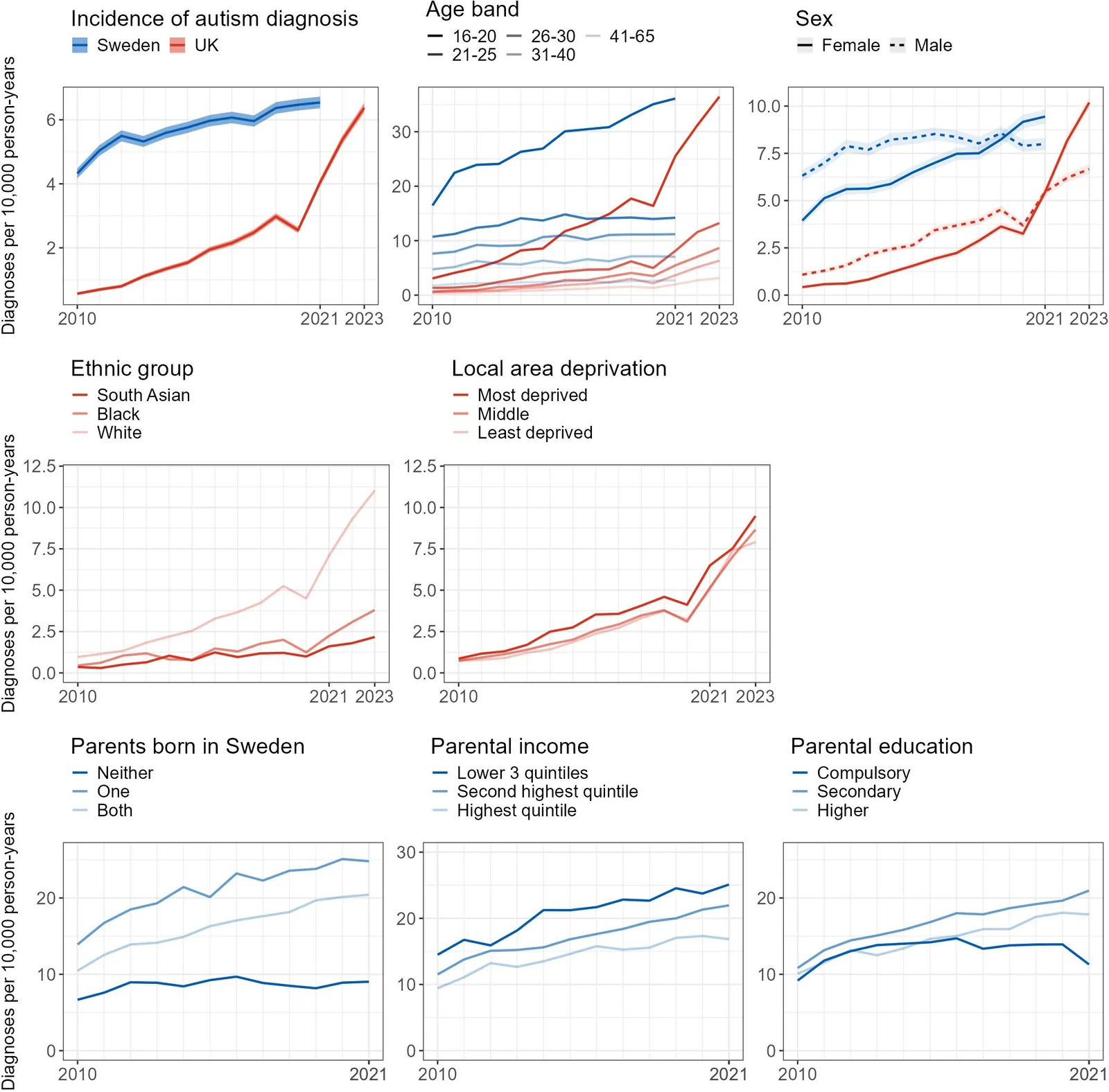
Top left: overall annual rates of first recorded autism diagnosis in the UK and Sweden. Top centre: annual rates of first recorded autism diagnosis by age group. Top right: annual age-standardised rates of first recorded autism diagnosis by sex. Middle row: annual age-standardised rates of first recorded autism diagnosis in the UK by ethnic group and postcode Index of Multiple Deprivation quintile. Bottom row: annual age-standardised rates of first recorded autism diagnosis in Sweden by parental birthplace, parental household income quintile at birth and parental education at age 15. For sex, ethnic group and IMD quintile, standardisation was performed using the mid-2022 age distribution of adults aged 16–65 years in the UK. For parental birthplace and education, the mid-2022 age distribution of adults aged 16–35 years in the UK was used due to limited information for over 35s. Shaded areas denote 95% CIs.

Both nations saw a divergence in rates when stratified by ancestral background. In the UK, rates of first recorded autism diagnosis increased faster over time in adults from white and mixed ethnic groups than black, South Asian or other ethnic groups. In Sweden, rates increased faster among adults with Swedish-born parents. These divergences were greater among females than males, as presented in online supplemental figure S1. Patterns by other socioeconomic variables were more nuanced. In the UK, rates of first recorded autism diagnosis were higher in the most deprived areas compared with the least deprived areas throughout the study period. But when stratified by sex, this pattern was only evident for males and not females by the end of the study period (online supplemental figure S1). In Sweden, throughout the study period, rates of first recorded autism diagnosis were higher among people who did not have a parent in the highest quintile of disposable income in their birth year. Sensitivity analyses demonstrated the same pattern for stratification by father’s income but not mother’s income (online supplemental figure S2). Rates of first recorded autism diagnosis increased more over time in people who had a parent completing upper secondary or higher education than people whose parents only completed compulsory education. This pattern was replicated in sensitivity analyses stratified by mother’s education level, but not when rates of first recorded autism diagnosis were stratified by father’s education level.

Predicted rates from Poisson regression models broadly fit those from the primary analysis (online supplemental figure S3). Statistical testing indicated that, on the multiplicative scale, there was strong evidence for a difference in the rate of increase in new recording of autism diagnoses between age groups in Sweden; between males and females in the UK and Sweden; and across categories of other measured sociodemographic variables, except for parental income level in Sweden (online supplemental table S8).

Trends were not explained by shifts in the completeness of variable recording. Across the study period, recording completeness was stable for IMD in the UK, gradually increased for ethnic group in the UK and slightly decreased over time for parental variables in Sweden (online supplemental figure S4). Rates of first-time autism diagnosis among the ‘unknown’ groups sat broadly in between the results of ‘known’ strata in the UK, but were lower than ‘known’ strata in Sweden (except for the ‘Neither Sweden’ group for parental birthplace; online supplemental figure S5). This was consistent with the fact that people with missing parental information were almost all born outside of Sweden, and autism diagnoses were lower in those born outside of Sweden (online supplemental figure S6).

For the analysis of neuropsychiatric history at the time of autism diagnosis, in the UK, 38 803 of the adults with a first recorded autism diagnosis were matched with 152 996 comparators; in Sweden, 48 496 adults with a first recorded autism diagnosis were matched with 188 421 comparators. Their sociodemographic characteristics are presented in online supplemental table S9 (online supplemental table S9). In the UK, autistic adults were more likely to be white and from a more deprived area than the comparators. In Sweden, autistic adults were more likely than comparators to have a parent who completed upper secondary education and have both parents born in Sweden. The distribution of observation time prior to the index date was stable across the study period in both nations (online supplemental figure S7). In Sweden, the median prior registration time was consistently higher among adults with first recorded autism diagnosis than matched comparators. This may be due to the autism group having a higher proportion of people born in Sweden than the comparator group (online supplemental able S9).

Trends in neuropsychiatric history prior to autism diagnosis are presented in figure 2 (conditions) and online supplemental figure S8 (medications), with underlying values in online supplemental table S10 and comparisons between adults diagnosed with autism in 2010 and those diagnosed in 2021 presented in online supplemental table S11. Sex-stratified results for conditions are presented in online supplemental figures S9 and S10. In both the UK and Sweden, more recently diagnosed autistic adults were less likely to have prior diagnoses of epilepsy, ID and psychotic disorders, or prescriptions/dispensations of sedatives than those diagnosed in earlier calendar years. These conditions did not become less common among the comparators during the study period. For example, in 2010, the age-standardised proportion of adults with a first recorded autism diagnosis who had prior records of epilepsy in the UK was 10% (95% CI 7.6% to 13%) compared with 4% (95% CI 3.6% to 4.5%) in 2023—the equivalent values were 1.3% (95% CI 0.8% to 1.8%) in 2010 and 1.2% (95% CI 1.1% to 1.4%) in 2023 for comparators.

**Figure 2:**
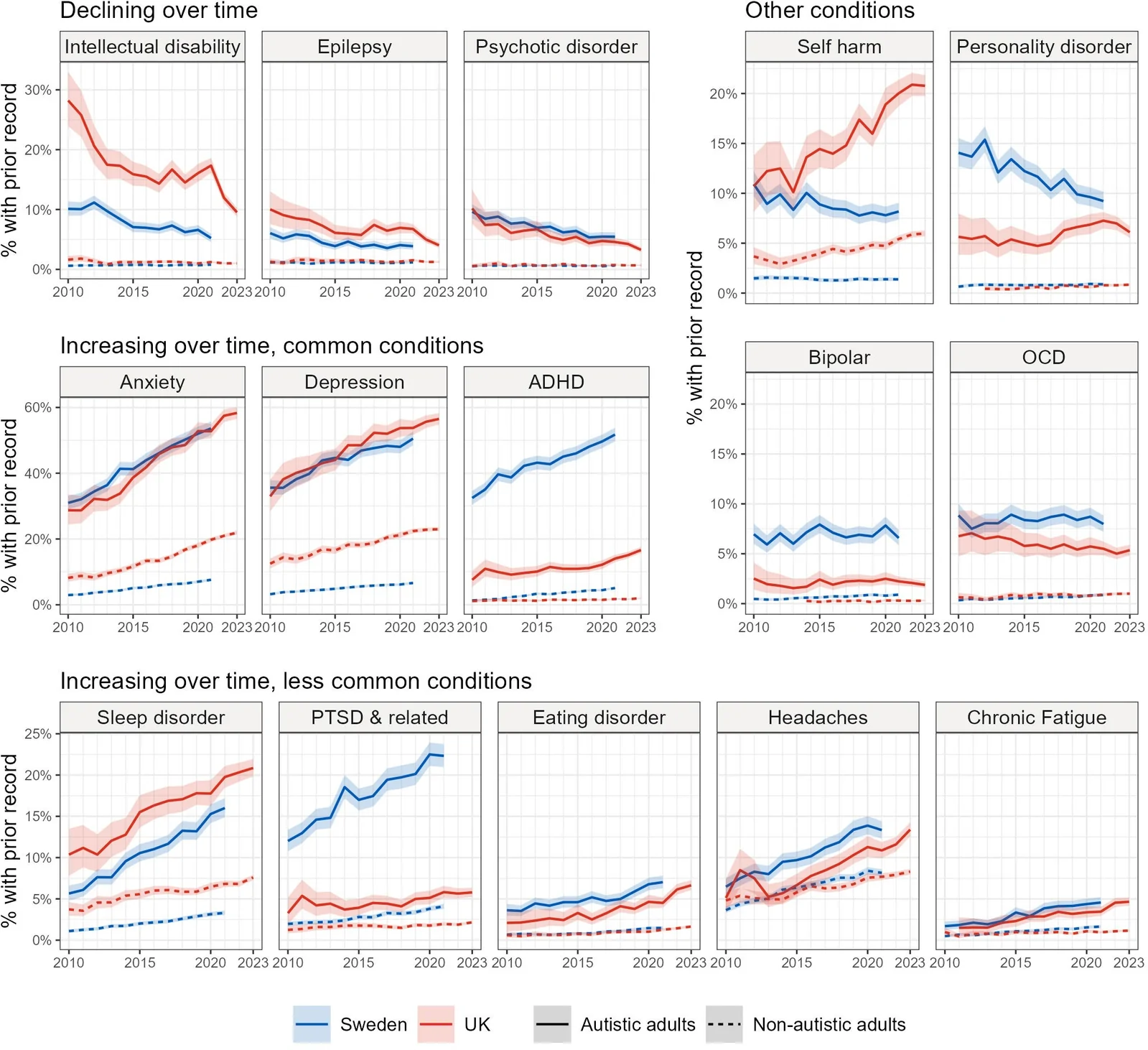
Age-standardised proportions of adults with a first recorded autism diagnosis, and matched non-autistic comparators, who had prior records of neuropsychiatric conditions, by year of autism diagnosis. Direct standardisation was performed using the empirical age distribution of adults with a first recorded autism diagnosis between 2010 and 2021 in UK and Swedish datasets using 5-year age bands from 16 years old to 65 years old. Shaded areas denote 95% CIs. ADHD, attention-deficit hyperactivity disorder; OCD, obsessive-compulsive disorder; PTSD, post-traumatic stress disorder.

In contrast, in both the UK and Sweden, more recently diagnosed autistic adults were more likely to have a prior record of anxiety, depression, ADHD, sleep disorders, eating disorders, stress-related disorders, headaches and chronic fatigue, as well as prior prescriptions/dispensation of antidepressants, melatonin, opioids and gabapentin than those diagnosed in earlier years (figure 2, online supplemental figure S8, online supplemental table S11). These conditions and medications were also more common over time in comparators, but the increases were lower in absolute magnitude. For example, the age-standardised proportion of adults with a first recorded autism diagnosis who had prior records of anxiety in the UK was 28.7% (95% CI 24.4% to 33.6%) in 2010 compared with 58.3% (95% CI 56.6% to 60.1%) in 2023. For comparators in the UK, the proportion with prior records of anxiety increased from 8.2% (95% CI 7% to 9.5%) in 2010 to 21.9% (95% CI 21.4% to 22.5%) in 2023. Trends were similar in direction between females and males; however, mental health conditions were generally more common for females than males, and the reduction in prior records of ID was greater in magnitude for females than males (online supplemental figures S9 and S10).

Several trends were inconsistent between the UK and Sweden. For example, adults with a first recorded autism diagnosis in the UK had an increasing probability over time of a prior record of self-harm but lower probability of prescriptions for antiseizure medications, antipsychotics and sedatives. These trends were not seen, and stable, in Sweden.

### All autistic adults (prevalent autism diagnoses)

The UK data included 24 537 039 adults with follow-up time between 2010 and 2023. The Swedish data included 9 096 874 adults with follow-up time between 2010 and 2021. The prevalence patterns for autism diagnoses by sociodemographic group are presented in figure 3, with underlying values in online supplemental tables S12–S14 and sex-stratified results in online supplemental figure S11. In both nations, age-standardised prevalence was consistently higher for males than females and increased in both groups over the study period to 1.4% for males and 0.7% for females in the UK in 2023, and to 1.8% for males and 1.2% for females in Sweden in 2021. In the UK, the male-to-female ratio decreased from 4.1 in 2010 to 2.2 in 2023. In Sweden, the male-to-female ratio decreased from 1.9 in 2010 to 1.6 in 2021. Prevalence increased over time in all age groups and was consistently higher in younger age groups. In the UK, the prevalence of autism in 16–20 year-olds was 4.1% in 2023; in Sweden, the prevalence of autism in 16–20 year-olds was 4.1% in 2021. As seen for rates of first recorded autism diagnosis, the prevalence for adults in the UK in the white ethnic group rose faster than the prevalence for adults in the black or South Asian ethnic groups, and the prevalence rose faster for adults with a parent born in Sweden than the prevalence for adults with neither parent born in Sweden. Patterns for other sociodemographic variables were also broadly consistent with those seen in analyses of first recorded autism diagnosis, including for sensitivity analyses (online supplemental figure S12).

**Figure 3:**
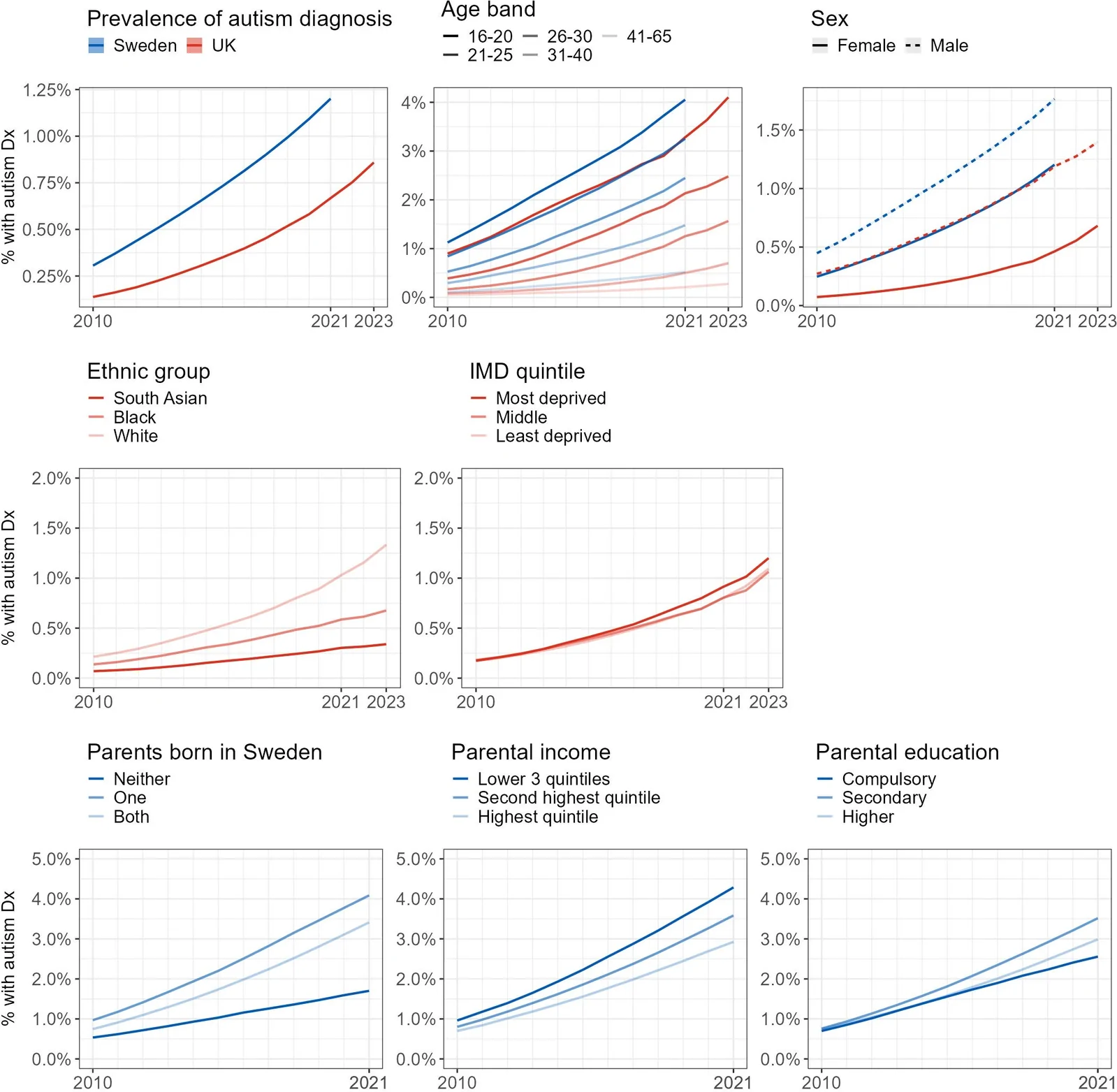
Top left: annual autism prevalence for adults in the UK and Sweden. Top centre: annual autism prevalence by age group. Top right: age-standardised annual autism prevalence by sex. Middle row: annual age-standardised autism diagnosis prevalence in the UK by ethnic group and postcode IMD quintile. Bottom row: annual age-standardised autism diagnosis prevalence in Sweden by parental birthplace, parental household income quintile at birth and parental education at age 15. For sex, ethnic group and IMD quintile, standardisation was performed using the mid-2022 age distribution of adults aged 16–65 years in the UK. For parental birthplace and education, the mid-2022 age distribution of adults aged 16–35 years in the UK was used due to limited information for over 35s. Shaded areas denote 95% CIs. Dx, diagnosis; IMD, Index of Multiple Deprivation.

The time trends for neuropsychiatric history of autistic adults are presented in figure 4 (conditions), online supplemental figure S13 (medications) and online supplemental table S15, with underlying values in online supplemental table S16 and sex-stratified analyses presented in online supplemental figures S13–S15. In both the UK and Sweden, changes over time and sex differences were consistently in the same direction as those seen in the analyses of newly diagnosed autistic adults, with decreasing ID, epilepsy and psychotic disorders, and increasing anxiety, depression, sleep disorders, ADHD, stress-related disorders, eating disorders, headaches, chronic fatigue, as well as use of antidepressants, melatonin, opioids and gabapentin. For example, the age-standardised proportions of autistic adults with prior records of epilepsy in the UK decreased from 14.6% (95% CI 13.9% to 15.3%) to 7.9% (95% CI 7.7% to 8.1%) in 2023, while the proportion with records of anxiety increased from 18.2% (95% CI 17.4% to 19%) to 44.3% (95% CI 43.8% to 44.7%). All conditions, except for ADHD and psychotic disorders, were more common in autistic females than autistic males in 2010. In both nations, many conditions, including anxiety, eating disorders and chronic fatigue, increased more among autistic females than autistic males over the study period, although sex differences generally grew faster in the UK than Sweden. On the other hand, ID and epilepsy decreased more among autistic females than autistic males; by the end of the study period, ID was more common in autistic males than autistic females in both nations.

**Figure 4:**
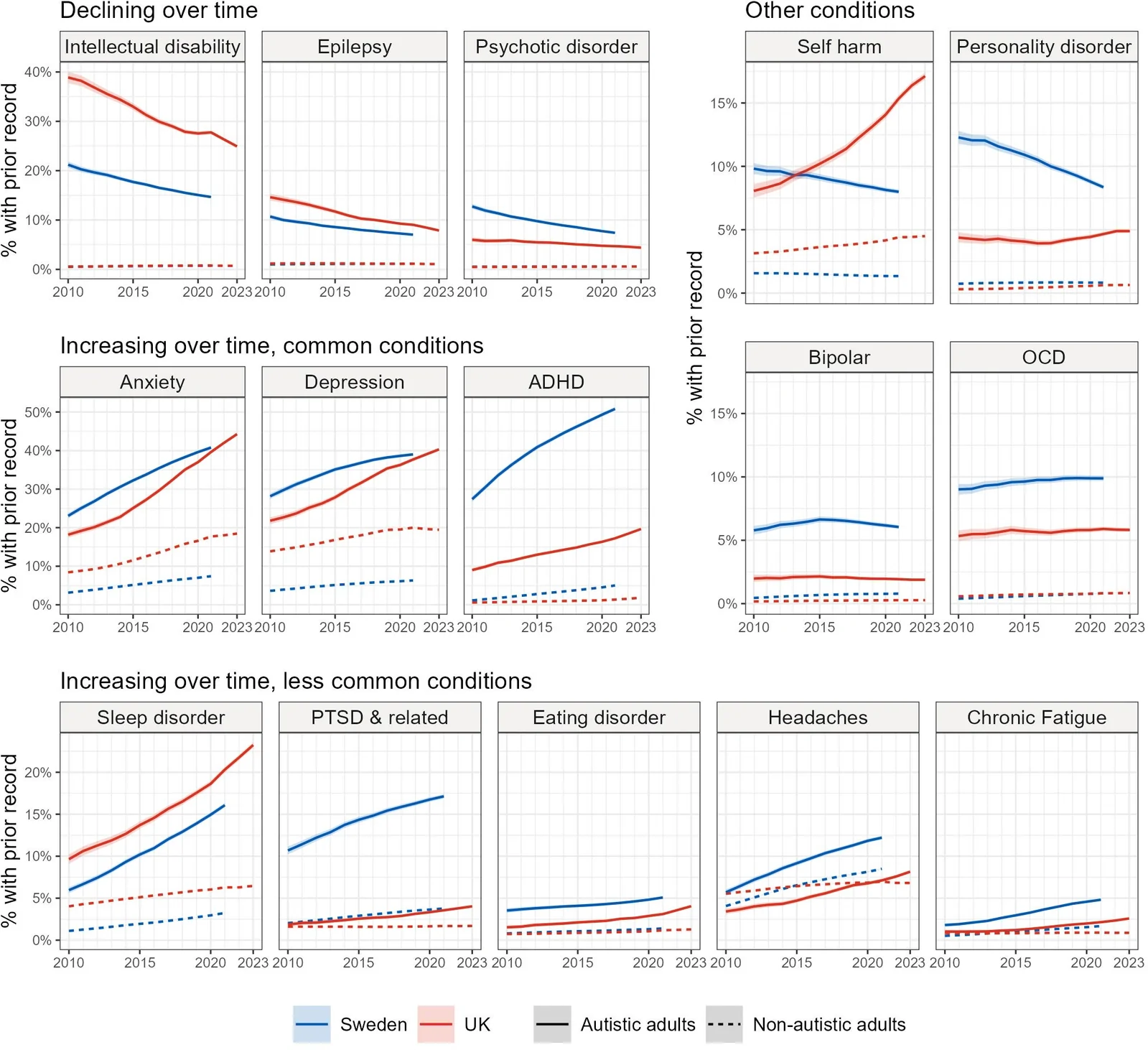
Annual age-standardised proportions of autistic adults and comparators with prior records of neuropsychiatric conditions. For standardisation, we used the empirical age distribution of autistic adults across the datasets in 2021, restricted to 16–65 year-olds. Shaded areas denote 95% CIs. ADHD, attention-deficit hyperactivity disorder; OCD, obsessive-compulsive disorder; PTSD, post-traumatic stress disorder.

## Discussion

This study found dramatic changes in the sociodemographic and neuropsychiatric profiles of adults diagnosed with autism in the UK and Sweden since 2010. In both nations, incident (first recorded) autism diagnoses have increased most among younger adults and females, and by the 2020s new diagnoses in adult females became more frequent than males. Prevalent autism diagnoses have also increased most among young adults, although prevalence remains higher among males than females across all adult age groups. Gaps in both incident and prevalent diagnoses have widened between ethnic groups in the UK and those with different parental backgrounds in Sweden, suggesting increasing inequality. Among both incident and prevalent autism diagnosis groups, there has also been a consistent decline over time in the proportions who have a history of ID, epilepsy and psychosis and an increase in the proportions with a history of depression, anxiety and several other neuropsychiatric conditions. Values for most of these background characteristics varied by sex; in the UK in particular, changes over time were generally larger in females than males.

### Comparison with other studies

A consistent increase over time in the prevalence of autism diagnoses has been reported in studies of registries and administrative records across the USA and Europe. In contrast, studies based on population surveys have shown features of autism to be stable over time in the UK^7^ and Sweden,^6^ being present in approximately 1% of the population. We found that approximately 4% of 16–20 year-olds had been diagnosed with autism in both the UK and Sweden at the end of the study period, suggesting that current diagnostic practice may have broadened since 2010 and be different from the criteria used in research settings. Administrative studies in the UK and Sweden have previously reported that the relative increase in diagnoses for females has been greater than for males, resulting in declining male-to-female ratios.^39 40^ In our current analysis, we have found that new autism diagnosis rates in adult females have overtaken those in adult males. Moreover, the male-to-female ratios of prevalent autism diagnoses at the end of the study period were 2.2 in the UK and 1.6 in Sweden—far lower than the 3.5:1 ratio estimated from population survey-based methods.^41^ The mechanism behind the widening departure from population survey-based estimates of the male-to-female ratio will need to be examined. For example, these findings may indicate an improved recognition of female presentations of autism, including the use of masking and camouflaging behaviours, addressing historic underdiagnosis. However, the possibility of other mental health presentations associated with camouflaging to be misidentified as autism, and in part contributing to the rapid increases in diagnosis rates among females, cannot be ruled out and may be a topic for future work.^42^ Indeed, the authors of the most widely used camouflaging questionnaire have recently cautioned against using the tool in making autism diagnostic decisions.^43^

Associations between SES and autism diagnosis rates have previously varied between contexts and the variables used to represent SES. US studies have previously reported lower autism prevalence in immigrant families^44^ and in black and Hispanic groups than non-Hispanic white groups.^16^ Ethnic minorities were also previously measured to have lower autism diagnoses rates in the UK^17^; in Sweden, autism without ID had a lower prevalence in immigrant families.^18^ In the USA, patterns by ethnic group appear to have reversed over time, and this has been interpreted as a sign of reducing inequalities in access to autism diagnoses within minority groups.^45^ For example, in California between 1992 and 2018, diagnoses increased at faster rates for children of black and Asian mothers.^46^ However, our findings indicate that diagnoses have increased faster for those in white ethnic groups in the UK and those with parents who were both Swedish born. This suggests that there may be cultural explanations to increased diagnoses in the UK and Sweden and potentially increasing ethnic disparities in healthcare access. Diagnostic patterns by other markers of SES appear more complex. Regarding other markers of SES, previous US studies have generally found more autism diagnoses in higher SES groups^14–16^ while the opposite has generally been found in other high-income nations.^17–21^ Our findings in Sweden suggest that families in which parents did not go beyond compulsory education have not had increases in autism diagnoses in line with the rest of the population. That said, autism prevalence is higher in more deprived areas in the UK and poorer families in Sweden. However, this socioeconomic patterning is no longer evident for new autism diagnoses in UK females; instead, similar diagnosis rates are seen between areas with different levels of deprivation. The reason for this is unclear but may suggest complex sex-specific dynamics that drive the process of seeking and receiving an autism diagnosis.

Previous studies in Sweden,^1^ the USA,^47^ Norway^48^ and Finland^19^ have reported autism diagnoses to have increased more for children without co-occurring ID than those with ID. Our findings show that, among adults with new autism diagnoses and those with lifetime autism diagnoses, there has been a decline in the proportion with a history of ID, epilepsy and psychosis, and these findings are consistent with previous studies. The present study also builds on previous findings in the UK that the increase in autism diagnoses in primary care records is mostly driven by terms that are presumed to describe autism without language or cognitive delays.^2^ Evidence from a Swedish twin study also showed that autism symptom scores in 7–12 year-olds diagnosed with autism in 2014 were lower than those diagnosed in 2004,^8^ and a meta-analysis of 11 meta-analyses found that the differences in neurocognitive constructs between autistic and non-autistic adults appear to have declined over time.^49^

A previous study in Sweden showed that more recently diagnosed autistic adults were more likely to have prior diagnoses of certain mental health conditions.^22^ Our work shows that these trends: (1) extend beyond mental health conditions to include certain medications, chronic fatigue and headaches; (2) are generalisable across countries and data sources; (3) are greater in magnitude than trends seen in adults without autism diagnoses; and (4) are also seen in adults with prevalent autism diagnoses. This latter finding could reflect changes in autism diagnostic practice and/or changes in the clinical needs of autistic adults, with relevance to public health planning either way. There are multiple potential explanations for the increase in mental health conditions over time. First, most of these conditions have also increasingly been diagnosed among adults without prior autism diagnoses in both our study and in national surveys^7 50^—we can expect autistic adults to also be subject to these temporal trends. Second, changes to diagnostic manuals mean that clinicians may be more willing to recognise co-occurring conditions and diagnose autism for those with other prior mental health conditions.^51^ Relatedly, it is possible that there is greater appreciation of nuanced forms of autism that may have previously been labelled as other conditions due to overlapping features, particularly in females.^10^ Conversely, a greater tendency to misattribute features that are typical of other conditions to autism cannot be ruled out. Together, the trends found in this study appear to reflect the changing context in which clinicians make diagnoses.^52^

### Strengths and limitations

Strengths of this study include the use of large prospectively recorded datasets that are broadly representative of the national population, reducing the risk of recall and selection bias and strengthening external validity. Also, consistent trends across two nations with different healthcare and recording practices enhance the external validity of findings. Similarly, identifying consistent trends among adults with both first-time autism records and prevalent autism records strengthens the plausibility that observed changes are relevant to the public health needs of adults with autism diagnoses and are accurately reflecting changes in diagnostic practice rather than being due to selection bias from changes in childhood diagnostic rates.

Some limitations of this study should be considered. We relied on the diagnoses and dates as recorded in the databases. Therefore, potential misclassification in these variables cannot be ruled out. In particular, the lack of a recorded diagnosis does not necessarily mean that someone has definitively not had that condition. This is a limitation common to all studies using electronic health records or registries. Given that we sought to understand the comparative trends in healthcare history of those diagnosed with autism over time rather than establish a fixed underlying estimate for the co-occurrence of neuropsychiatric conditions, such misclassification may not be a fundamental concern. Also, any misclassification of diagnosis dates is unlikely to affect the direction of trends observed over the long study period. Relatedly, we started the study period in 2010 rather than earlier, allowing a 9-year lead time after the addition of Swedish outpatient records to the NPR, to increase the chances that a first recording of autism truly represented a new diagnosis, rather than re-recording of a diagnosis made before 2001. Another limitation was that data for Sweden beyond 2021 were unavailable, and certain findings in the UK were particularly evident after 2021, such as the decline in prior antiseizure and antipsychotic medication prescriptions. Furthermore, the underlying healthcare systems and data sources in the UK and Sweden are different, and so variation in results between nations may reflect differences in healthcare systems and data capture rather than true differences in diagnostic patterns. For example, the UK data were derived from primary care records, whereas the Swedish registers primarily cover secondary care records. This may explain the contrasting trends in the results for self-harm in the two datasets: in the UK, self-harm was captured if the general practitioner had a record of this, whereas in Sweden it had to be recorded during an inpatient episode. Additionally, postcode-based IMD scores in England and individual-level parental background measured in Sweden are different sociodemographic constructs and so may not be directly comparable. Nevertheless, the consistency of majority of the results across the two nations was reassuring.

### Clinical implications

The changes observed in this study have implications for planning mental health and social care support. The reasons behind the observed changes therefore need further discussion and scrutiny. First, autism diagnoses have not increased as much in ethnic minority or immigrant groups, and so the mechanisms behind such disparities will need to be considered. Second, the high rates of previously diagnosed mental health conditions in individuals diagnosed with autism in recent years highlight the complexity in current autism diagnostic practice. Diagnostic services may need to have the skills to consider broader psychiatric formulation, including considering other diagnoses as an alternative or in addition to the autism diagnosis. This would contrast with the current siloed services, particularly in the UK, which are often commissioned to focus on just an autism diagnosis, and may rely on remote online methods that potentially limit assessment quality.^53^ Finally, it is clear that the distribution of co-occurring conditions associated with autism has changed significantly over time. While these data are unable to shed light on whether these changes indicate greater appreciation of nuanced forms of autism, or misdiagnoses of other psychiatric presentations as autism, efforts will need to be made to recognise and address the needs with appropriate support.

In conclusion, the sociodemographic and neuropsychiatric backgrounds of adults diagnosed as autistic have changed dramatically since 2010. The reasons for these changes need to be investigated in future research coproduced in partnership with the autistic community. It will be essential to do this while prioritising the well-being of autistic people and addressing health disparities in this population.

## Supplementary material

10.1136/bmjment-2026-302983 Supplementary file 1

10.1136/bmjment-2026-302983 Supplementary file 2

10.1136/bmjment-2026-302983 Supplementary file 3

## Data Availability

Data may be obtained from a third party and are not publicly available.
