# Supplementary material for: Changes in the profile of adults diagnosed as autistic since 2010: population-based studies in the UK and Sweden
Source: BMJ Ment Health. 2026 Nov 3;29(1):e302983. doi: 10.1136/bmjment-2026-302983 (PMC13735358; doi:10.1136/bmjment-2026-302983)
Supplement: Supplementary file 1 [file bmjment-29-1-s001.docx]

# Supplementary Methods

We statistically evaluated sociodemographic differences in trends of new records of autism diagnosis on the multiplicative scale, through interaction testing. Separately for each sociodemographic variable, we calculated the total number of first recorded autism diagnosis and total eligible follow-up time, by sociodemographic group, age and calendar year. In line with the primary analyses, we limited the data to 16-65 year-olds for analysis of age groups, sex, UK ethnicity, and UK IMD. We limited the data to 16-35-year-olds for analyses of parental variables in Sweden. We fit Poisson regression models for counts of first recorded autism diagnoses, with fixed effects of: calendar year (centred at 2015), sociodemographic group, an interaction term between calendar year and sociodemographic group, and an offset term of the logarithm of follow-up time. For all sociodemographic variables except for age group, the models also included the effect of natural splines of age with 3 degrees of freedom. Although the effect of calendar year may be more closely fit by non-linear terms, it was treated as a linear effect to allow clearer interpretation of interaction terms. A natural spline of age with 3 degrees of freedom was chosen to allow the effect of age to vary with a high degree of flexibility. To visually assess model fit, we predicted the annual rate of new record of autism diagnosis by sociodemographic group, with weighting by age, based on the same standard population used in primary analyses, and then overlayed the model-predicted values over those from the primary analysis. The model parameters for the interaction terms in each model were presented, alongside p-values. A positive interaction term indicates that, on the multiplicative scale, there is a greater increase over time in the rate of first records of autism diagnosis for that sociodemographic group compared to the reference sociodemographic group.
