# Supplementary material for: Changes in the profile of adults diagnosed as autistic since 2010: population-based studies in the UK and Sweden
Source: BMJ Ment Health. 2026 Nov 3;29(1):e302983. doi: 10.1136/bmjment-2026-302983 (PMC13735358; doi:10.1136/bmjment-2026-302983)
Supplement: Supplementary file 2 [file bmjment-29-1-s002.docx]

# Supplementary figures

**Figure S1:** Top left: annual rate of first recorded autism diagnosis by age group and sex in the UK. Top centre left: annual age-standardised rate of first recorded autism diagnosis in the UK, by ethnic group and sex. Top centre right: annual age-standardised rate of first recorded autism diagnosis in the UK by postcode-level index of multiple deprivation quintile. Bottom left: annual rate of first recorded autism diagnosis by age group and sex in the UK. Bottom centre left: annual age-standardised rate of first recorded autism diagnosis in Sweden, by parental birthplace and sex. Bottom centre right: annual age-standardised rate of first recorded autism diagnosis in Sweden, by parental household income quintile and sex. Bottom right: annual age-standardised rate of first recorded autism diagnosis in Sweden, by parental education and sex. For sex, ethnic group and local area deprivation quintile, standardisation was performed using the mid-2022 age-distribution of 16-65 year old adults in the UK. For parental birthplace and education, the mid-2022 age-distribution of 16-35 year old adults in the UK was used, due to limited information for over 35s.

**Figure S2:** First column: among 16-35y/o adults in Sweden with follow-up in 2021, the distribution of paternal household income quintile at birth (top row), maternal household income quintile at birth (second row), paternal education level age index person’s 15^th^ birthday (third row), maternal education level age index person’s 15^th^ birthday (bottom row). Middle column: annual age-standardised rate of first recorded autism diagnosis in Sweden, by the sociodemographic strata described in first column. Standardisation was performed using the mid-2022 UK age-distribution of 16-35 year old adults, as parental linkage was unavailable for people born before 1973. Shaded areas = 95% confidence intervals.

**Figure S3:** Comparison of model-based predictions of annual rates of first recorded autism diagnosis, to rates the that were calculated in primary analyses and presented in Figure 1. Black lines are the predictions from Poisson regression models and coloured lines are the estimates reported in the main analyses.

**Figure S4:** Percentage of total follow-up time in each calendar year, in the analysis of first recorded autism diagnosis, that was from people in each sociodemographic stratum of Index of Multiple Deprivation (IMD) and ethnic group in the UK, and parental background variables in Sweden. Dashed lines represent people with unknown values for the variable in question.

**Figure S5:** Annual rates of first records of autism diagnosis, for strata of Index of Multiple Deprivation (IMD) and ethnic group in the UK, and parental background variables in Sweden. Rates for people with unknown values for each variable are presented as grey lines.

**Figure S6:** Plots to better understand the group with missing parental birthplace information in Sweden. Top row: Among all people eligible to contribute to the study, the percentage of people in the unknown parental birthplace group who were born in Sweden themselves, or not. Second row: The annual percentage of people who have unknown parental birthplace, by own birthplace. Third row: Annual age-standardised rates of first records of autism diagnosis, by own person’s birthplace. Bottom row: Annual age-standardised prevalent autism diagnosis rate, by own person’s birthplace.

**Figure S7:** Median and interquartile range of years of prior registration before the index date, for adults with a first record of autism and matched non-autistic comparators, by country and calendar year.

**Figure S8:** Top row: Age-standardised proportions of newly diagnosed autistic adults with prior records of neuropsychiatric medications, by year of autism diagnosis. Middle row: Age-standardised proportions of newly diagnosed autistic adults in the UK with prior records of neuropsychiatric medications, by sex and by year of autism diagnosis. Bottom row: Age-standardised proportions of newly diagnosed autistic adults in Sweden with prior records of neuropsychiatric medications, by sex and by year of autism diagnosis. Direct standardisation was performed using the empirical age-distribution of adults newly diagnosed with autism across the study period in UK and Swedish datasets, using five-year age bands from 16 to 65 years old. Shaded areas = 95% confidence intervals.

**Figure S9:** Age-standardised proportions of newly diagnosed autistic adults in the UK with prior records of neuropsychiatric conditions, by sex and by year of autism diagnosis. Direct standardisation was performed using the empirical age-distribution of adults newly diagnosed with autism across the study period in UK and Swedish datasets, using five-year age bands from 16 to 65 years old. Shaded areas = 95% confidence intervals. ADHD = attention deficit hyperactivity disorder.

**Figure S10:** Age-standardised proportions of newly diagnosed autistic adults in Sweden with prior records of neuropsychiatric conditions, by sex and by year of autism diagnosis. Direct standardisation was performed using the empirical age-distribution of adults newly diagnosed with autism across the study period in UK and Swedish datasets, using five-year age bands from 16 to 65 years old. Shaded areas = 95% confidence intervals. ADHD = attention deficit hyperactivity disorder.

**Figure S11:** Top left: annual prevalence of autism diagnosis by age group and sex in the UK. Top centre left: annual age-standardised prevalent autism diagnosis rate in the UK, by ethnic group and sex. Top centre right: annual age-standardised prevalent autism diagnosis rate in the UK by postcode-level index of multiple deprivation quintile and sex. Bottom left: annual prevalence of autism diagnosis by age group and sex in Sweden. Bottom centre left: annual age-standardised prevalent autism diagnosis rate in Sweden, by parental birthplace and sex. Bottom centre right: age-standardised prevalent autism diagnosis rate in Sweden, by parental income quintile and sex. Bottom right: annual age-standardised prevalent autism diagnosis rate in Sweden, by parental education and sex. For sex, ethnic group and local area deprivation quintile, standardisation was performed using the mid-2022 age-distribution of 16-65 year old adults in the UK. For parental birthplace and education, the mid-2022 age-distribution of 16-35 year old adults in the UK was used, due to limited information for over 35s.

**Figure S12:** Annual age-standardised autism diagnosis prevalence in Sweden, by paternal income quintile in index person's birth year (top row), maternal income quintile in index person's birth year (second row), paternal education level when index person turned 15 (third row), and maternal education level when index person turned 15 (bottom row). Overall (left column) & sex-stratified (right column). Standardisation was performed using the mid-2022 UK age-distribution of 16-35 year old adults, as parental linkage was unavailable for people born before 1973. Shaded areas = 95% confidence intervals.

**Figure S13:** Top row: Age-standardised proportions of newly diagnosed autistic adults with prior records of neuropsychiatric medications, by year of autism diagnosis. Middle row: Age-standardised proportions of newly diagnosed autistic adults in the UK with prior records of neuropsychiatric medications, by sex and by year of autism diagnosis. Bottom row: Age-standardised proportions of newly diagnosed autistic adults in Sweden with prior records of neuropsychiatric medications, by sex and by year of autism diagnosis. Direct standardisation was performed using the empirical age-distribution of adults newly diagnosed with autism across the study period in UK and Swedish datasets, using five-year age bands from 16 to 65 years old. Shaded areas = 95% confidence intervals.

**Figure S14:** Age-standardised proportions of autistic adults in the UK with prior records of neuropsychiatric conditions, by sex and by year of autism diagnosis. For standardisation we used the empirical age-distribution of autistic adults across the datasets in 2021, restricted 16-65 year olds. Shaded areas = 95% confidence intervals. ADHD = attention deficit hyperactivity disorder.

**Figure S15:** Age-standardised proportions of autistic adults in Sweden with prior records of neuropsychiatric conditions, by sex and by year of autism diagnosis. For standardisation we used the empirical age-distribution of autistic adults across the datasets in 2021, restricted 16-65 year olds. Shaded areas = 95% confidence intervals. ADHD = attention deficit hyperactivity disorder.
